# Pulmonary Complications in Cirrhotic Candidates for Liver Transplantation

**Published:** 2010-06-01

**Authors:** Seiyed Mohammad Ali Ghayumi, Samrad Mehrabi, Mahmood Zamirian, Javad Haseli, Kamran Bagheri Lankarani

**Affiliations:** 1 Department of Internal Medicine, Nemazee Hospital, Shiraz University of Medical Sciences, Shiraz, Iran; 2 Department of Cardiology, Nemazee Hospital, Shiraz University of Medical Sciences, Shiraz, Iran; 3 Faculty of Medicine, Shiraz University of Medical Sciences, Shiraz, Iran

**Keywords:** Cirrhosis, Portopulmonary Hypertension, Hypoxemia, Alveolar-Arterial Oxygen Difference

## Abstract

**Background and Aims:**

The determination of the prevalence of cardiopulmonary complications at a liver transplant center in Iran.

**Methods:**

Ninety-nine patients (61 male and 38 female) with a mean age of 36.5 (15-66) years with proven cirrhosis were enrolled in this study. Patients with primary cardiac disease, current smokers, those with sepsis, hepatocellular carcinoma, recently ruptured esophageal varices and chronic pulmonary or renal diseases were excluded from the study. Sixty-nine patients had ascites. Forty-four patients had grade C Child-Pugh classification. All patients were evaluated for respiratory function by chest X-ray (CXR), room air arterial blood gas, simultaneous pulse oximetry, cardiac echocardiography and spirometry.

**Results:**

Sixty-one patients (66.1%) had a widened alveolar-arterial O2 difference ( > 20 mmHg); 14 (14.1%) had hypoxemia; 6 (6.1%) had mean pulmonary arterial pressure (MPAP) = 25-40 mmHg; 12 (12.1%) had tricuspid regurgitation; pleural effusion and lung restriction were detected in 4 (4%) and 50 (50.5%), respectively. P(A-a)O2 was negatively associated with pulmonary hypertension (P < 0.03) and tricuspid regurgitation (P < 0.005). Portal hypertension and portal vein thrombosis were detected in 91 and 8 patients, respectively.

**Conclusions:**

A widened alveolar-arterial oxygen difference was common in our patients, but hypoxemia occurred in 14% of patients. Portopulmonary hypertension was preponderant in those patients of male gender.

## Introduction

Patients with cirrhosis develop complications in a variety of organs including the heart, lungs, kidneys, and other organ systems. A variety of causes for cardiac and pulmonary dysfunction in liver disease have been identified and include intrinsic cardiopulmonary disorders not specifically related to liver disease, as well as unique problems associated with the presence of liver disease and/or portal hypertension.

Owing to the success of orthotopic liver transplantation (OLT), there has been increasing recognition of the importance of the cardiac and pulmonary vascular complications of hepatic disease states.

Pulmonary complications include hepatopulmonary syndrome, hepatic hydrothorax, and pulmonary hypertension [1][2][3]. In screening studies of patients with chronic liver disease, arterial blood gas (ABG) abnormalities are found in as many as 45% of patients, and abnormal pulmonary function tests in as many as 50% [4].

Cardiac complications are hemodynamic changes, such as increased cardiac output and regional organ blood flow, decreased arterial pressure, and low peripheral vascular resistance [5].

This study reports the frequency of pulmonary complications in a population of cirrhotic patients undergoing preoperative liver transplant evaluation.

## Materials and Methods

### Patients

In a cross-sectional study, ninety-nine consecutive patients (61 male, 38 female, age 15-66, mean age 39.65 ± 11.65) with histologically proven cirrhosis, who were on a liver transplantation waiting list at Shiraz Transplant Center, were evaluated. Exclusion criteria were: recently ruptured esophageal varices, primary cardiac disease, chronic bronchopulmonary disease, renal disease, sepsis, current smoking and hepatocellular carcinoma. The etiologies of cirrhosis were: viral in 38 (hepatitis B:34, hepatitis C:4); cryptogenic in 26; autoimmune hepatitis in 13; primary sclerosing cholangitis in 12; and other less frequent causes in 10 patients. The severity of cirrhosis was assessed according to the Child-Pugh classification. There were 8 grade A (8.1%), 47 grade B (47.5%) and 44 grade C (44.4%) patients. Portal hypertension was detected in 91 patients, and portal vein thrombosis in 8 patients.

### Meseaurments

Arterial blood gas, pulse oximetry, chest radiography, doppler echocardiography, doppler sonography of portal vein, spirometry and liver function tests were performed on all patients. Hypoxemia was considered when PaO2 < 70 mmHg. Mean pulmonary arterial pressure (MPAP) was calculated based on the tricuspid regurgitation gradient; and pulmonary hypertension was considered when MPAP > 25 mmHg, and portal hypertension was defined as flow velocity < 20 cm/s by doppler sonography.

### Statistical analysis

The results are expressed as mean ± standard deviation. The chi-square test, Fisher exact test andthe bivariate correlation test were used, where appropriate, to analyze the data provided. SPSS ver. 11.50 was used for statistical analysis. Changes were reported as significant if P < 0.05.

## Results

### Hypoxemia and alveolar-arterial oxygen pressure difference

Sixty-one patients (66.1%) had a widened alveolar-arterial oxygen difference (> 20 mmHg) (41 male and 20 female, mean age 40.36); 42 (73.8%) had ascites. The proportion of patients with pulmonary hypertension in the widened P (A-a) O2 group (1/61) was significantly lower than in the normal P(A-a)O2 group (5/38) (P = 0.03). Fifty-four percent of patients with abnormal P(A-a)O2 had lung restriction. All of the hypoxemic patients (14.1%) were in the abnormal P (A-a) O2 group (P = 0.001). The proportion of patients with tricuspid regurgitation in the normal P(A-a)O2 group (9 /38) was greater than in the abnormal group (3/61) ( P = 0.009). There was a significant positive correlation between P(A-a)O2 and prothrombin time (P = 0.03)(r= 0.274).

A widened P(A-a)O2 has no association with the etiology and severity of cirrhosis, age, sex, blood group, ascites, lung volume, portal hypertension, portal vein thrombosis, serum albumin and bilirubin.

The proportion of patients with hypoxemia was significantly greater in the Child-Pugh grade C patients(11/44), than in the Child-Pugh grade B patients(3/47) or in the Child-Pugh grade A patients (0/8) (P = 0.019). None of the hypoxemic patients had pulmonary hypertension or pleural effusion.

The mean PaCO2 were 35.06 ± 4.54 which was lower in those patients with abnormal P(A-a)O2 (32.86 ± 4.91 vs. 36.50 ± 5.51)(table 1).

**Table1:** General characteristics in cirrhotic patients with normal and abnormal P(A-a)O2

**Child-Pugh**	**Age(y/o)**	**Normal P(A-a)O2**	**Abnormal P(A-a)O2**	**P value**
		**38.5 ± 12.19**	**40.36 ± 11.51**	**0.44**
A	2	6	
B	21	26	
C	15	29	
Gender	Male	20	41	
	Female	18	20	
Ascites		27	42	0.87
Pulmonary hypertension		5	1	0.03^a^
Portal hypertension		37	54	0.14
Portal vein thrombosis		3	5	0.85
Tricuspid regurgitation		9	3	0.009^a^
Serum Albumin(mg/dL)		3.33 ± 0.56	3.34 ± 0.65	0.93
Serum Bilirubin(mg/dL)		3.23 ± 2.77	2.83 ± 1.78	0.38
Prothrombin Time(sec)		15.27 ± 2.04	16.60 ± 2.93	0.016^a^
pH		7.39 ± 0.064	7.41 ± 0.047	0.076
PaCO2 (mmHg)		36.50 ± 5.51	32.86 ± 4.91	0.0009^a^
PaO2 (mmHg)		88.99 ± 5.23	76.98 ± 9.08	0.0001^a^

^a^ Significance of differences between groups: P < 0.05

### Lung restriction

Fifty patients in this study had lung restriction (32 male, 18 female, mean age 40.26) out of which 24 (48%) showed mild, and 14 (28%) tense, ascites and 12 (24%) were without ascites. This showed no significant association between lung restriction and ascites, except for tense ascites (P = 0.04).

Thirty patients (66%) with restrictive lung disease had widened P(A-a)O2 without significant association. There was no significant association between lung restriction and the severity and etiology of cirrhosis, sex, blood group, portal hypertension, portal vein thrombosis, bilirubin and prothrombin time. There was a significant correlation between pleural effusion and moderate or severe lung restriction (6) (P = 0.033).

### Pulmonary hypertension

Six patients (6.1%) (4 male, 2 female) had MPAP > 25 mmHg, 4 in Child-Pugh grade B and 2 in Child-Pugh grade C, without right atrial or ventricular hypertrophy or dilatation. All of them had portal hypertension, and 4 patients had tricuspid regurgitation (P = 0.002). The etiology of the cirrhosis was cryptogenic in 4 (67%) (P = 0.04). Pulmonary hypertension had no significant association with the severity of cirrhosis, sex, ascites and hypoxemia (table 2).

**Table2:** General characteristics in patients with and without PPHTN.

**Age**		**PPHTN^b^**	**No PPHTN**	**P value**
		**39.5 ± 9.87**	**39.66 ± 11.91**	**0.88**
Sex	Male	4	57	0.57
	Female	2	36	
Child-Pugh classification	A	0	8	0.002^a^
	B	4	43	
	C	2	42	
Ascites	Negative	0	34	0.67
	Mild	4	52	
	Tense	2	17	
Spirometry	No restriction	4	45	0.33
	Mild restriction	2	26	
	Moderate restriction	0	18	
	Severe restriction	0	4	
P(A-a)O2		15.94 ± 8.09	26.21 ± 11.22	0.03^a^
Hypoxia		0	14	0.59
Tricuspid regurgitation		4	8	0.002^a^

^a^ Significance of differences between groups: P < 0.05

^b^ PPHTN: Portopulmonary hypertension

### Pleural effusion

Pleural effusion was detected in 4 patients (4%) (3 of them male), 2 right-sided, 1 left-sided, and 1 bilateral. The patients with pleural effusion were younger (32.75 ± 7.76 vs. 39.94 ± 11.83) and 75% had widened P(A-a)O2, with significant association with tense ascites (P = 0.033).

### Tricuspid regurgitation

Twelve patients (12.1%), (7 male, 5 female; mean age 37.66) had tricuspid regurgitation (TR), 7 in Child-Pugh grade B and 5 in Child-Pugh grade C. 8 of them had ascites and11 had portal hypertension. None of these patients had hypoxemia. Pleural effusion was detected in these groups of patients (16.7%) more than in patients without TR (2.3%). The proportion of patients with TR in the pulmonary hypertensive group (4/6) was significantly greater than in patients with normal pulmonary arterial pressure (8/93) (P < 0.001).

## Discussion

Most patients in this study showed one or more types of cardiopulmonary complications. Pulmonary complications were: widened alveolar-arterial oxygen difference; ventilatory restriction; and hepatic hydrothorax. Cardiovascular complications were: pulmonary hypertension; and tricuspid regurgitation. The most common pulmonary complication in this study was widened P(A-a)O2(61.5%), which was four times greater than hypoxemia; this may be related to pulmonary arteriovenous shunting, portopulmonary shunting, and intrapulmonary vascular abnormalities, limited diffusion of oxygen, and/or ventilation-perfusion mismatching [7]. Different reports have given different frequencies of reduced arterial oxygen saturation in cirrhotic patients varying from about 10% to as high as 70% [8].In a prospective study by Vachiery et al. [9], 14% of 120 cirrhotic patients had a PaO2 of < 70 mmHg, and this hypoxemic subset was characterized by more severe liver failure with a higher Child-Pugh score and a higher proportion of grade C disease. In another study by Yigit et al. [10] the frequency of hypoxia was found to be 33.3%, with the same threshold of hypoxia, which is markedly more than in our study (14%),which might be the result of different characteristics of patient  populations.

There was a greater frequency of alveolar- arterial oxygen difference than hypoxemia, since normoxemia can be maintained by alveolar hyperventilation, even with a widened P(A-a)O2.

In our study, there was widened P(A-a)O2 in 66% of patients, and hypoxia in 14%. There was no significant difference in Child–Pugh grade between the abnormal P(A-a)O2 group and the normal P(A-a)O2 group, indicating that the severity of cirrhosis does not play a major role in the development of P(A-a)O2, but hypoxemia occurs more in Child–Pugh grade C. This finding suggests that the widened P(A-a)O2 occurs in the early stages of cirrhosis, with the same frequency as in the late stages, but that hypoxemia occurs mostly in the late stages of cirrhosis. This is consistent with previous reports [7][9][11][12][13].

In their study, Krowka et al. [14]reported that there was no significant relationship between hypoxia and biochemical parameters (PT, albumin, bilirubin, and aminotransferases) of liver function. Similarly, there were no significant differences between the biochemical parameters of liver function and hypoxia in our patients, except for a positive correlation between P(A-a)O2 and prothrombin time (P = 0.03)(r= 0.274).

Different reports have given different frequencies of ventilatory restriction in cirrhotic patients, varying from about 9% [15][16] to as high as 24% [17], which may be the result of interstitial lung edema, ascites, and pleural fluid. In our study, 50 patients (50.5%) showed ventilatory restriction, significantly associated with tense ascites (P = 0.04).

Since patients with advanced liver disease usually hyperventilate, hypocapnia (PaCO2<35 mmHg) and respiratory alkalosis are common [13][18][19]. In our study most patients had respiratory alkalosis, which was greater in the subgroup of patients with widened P(A-a)O2.

Portopulmonary hypertension (PPHTN) is an uncommon, but serious complication of cirrhosis, and is associated with a grave prognosis. The incidence of PPHTN has been reported to be in a range of 0.25% to 6% [8][20]. McDonnell et al. [21] reviewed the results of 17,901 autopsies and found that primary pulmonary hypertension occurred in 0.13% of all patients but in 0.73% of patients with cirrhosis. In another study by Benjaminov et al. [22], the prevalence of PPHTN in cirrhotic patients with refractory ascites was found to be 16.1%. In the present study, the prevalence of moderate PPHTN(25-40 mmHg) in patients with cirrhosis was 6%, which is lower than the results in the study by Castro et al. [23]; it occurs in patients with portal hypertension, which is consistent with previous reports [24][25].

In agreement with previous studies [18][22][25][26].there was no correlation between PPHTN and the severity of liver disease as assessed by the Child-Pugh score.

A moderate male predominance was found in our study: 1:0.8 (male to female), as has been found by others [25][27][28] and in contrast to the general epidemiology of pulmonary hypertension [29].This suggests that factors other than female hormones may contribute to portopulmonary hypertension [30].

Another interesting result was the finding of a higher proportion of portopulmonary hypertension in cryptogenic cirrhosis, compared with patients with other types of cirrhosis, 4 out of 6 patients (P = 0.04).

## Conclusions

A widened alveolar-arterial oxygen gradient was detected in 61% of patients, four times more often than hypoxia, the latter being associated with the severity of liver disease. In the present study, the prevalence of PPHTN in cirrhotic patients was found to be 6%, with a male-to-female ratio of 1.25, and a significant positive correlation with cryptogenic cirrhosis (P = 0.04).
